# The Aβ_42_:Aβ_40_ ratio modulates aggregation in beta-amyloid oligomers and drives metabolic changes and cellular dysfunction

**DOI:** 10.3389/fncel.2024.1516093

**Published:** 2024-12-05

**Authors:** Annika Haessler, Stefanie Gier, Nathalie Jung, Maike Windbergs

**Affiliations:** Institute of Pharmaceutical Technology, Goethe University Frankfurt, Frankfurt am Main, Germany

**Keywords:** Alzheimer’s disease, Aβ oligomers, blood–brain barrier, Raman microscopy, lipid droplets, Aβ_42_:Aβ_40_

## Abstract

The pathophysiological role of Aβ_42_ oligomers in the onset of Alzheimer’s disease (AD) is heavily disputed, pivoting research toward investigating mixed oligomers composed of Aβ_42_ and Aβ_40_, which is more abundant but less aggregation-prone. This study investigates Aβ_42_:Aβ_40_ oligomers in different ratios, examining their adverse effects on endothelial cells, neurons, astroglia, and microglia, as well as in a human blood–brain barrier (BBB) model. Combining label-free Raman microscopy with complementary imaging techniques and biochemical assays, we show the prominent impact of Aβ_40_ on Aβ_42_ fibrillation, suggesting an inhibitory effect on aggregation. Mixed oligomers, especially with low proportions of Aβ_42_, were equally detrimental as pure Aβ_42_ oligomers regarding cell viability, functionality, and metabolism. They also differentially affected lipid droplet metabolism in BBB-associated microglia, indicating distinct pathophysiological responses. Our findings demonstrate the overarching significance of the Aβ_42_:Aβ_40_ ratio in Aβ oligomers, challenging the traditional focus on Aβ_42_ in AD research.

## 1 Introduction

Alzheimer’s disease (AD) is a neurodegenerative disorder characterized by extracellular deposition of aggregated beta-amyloid (Aβ) peptides, so-called Aβ plaques, and buildup of intracellular hyperphosphorylated tau, both triggering neurodegeneration (DeTure and Dickson, 2019). Although Aβ and tau are considered hallmarks, the past decades of research were mainly steered by the amyloid hypothesis, highlighting Aβ aggregation as the cause of AD (Krafft et al., 2022). However, this postulation has been repeatedly challenged since numerous therapeutic approaches focusing on Aβ plaques have not led to a significant improvement in the disease (Zhang et al., 2023). Moreover, Aβ aggregation and plaque formation start years before symptomatic onset, thus, these Aβ-targeting treatments may also simply be too late to effectively cure AD (Zhang et al., 2023). Therefore, the amyloid hypothesis has been revised, identifying soluble Aβ oligomers, rather than Aβ plaques, as the primary toxic amyloid type (Vadukul et al., 2020; Deleanu et al., 2022; Krafft et al., 2022). While ample evidence underlines the harmful effect of Aβ oligomers, therapeutic developments are still inadequate, and thus, their actual toxicity is heavily discussed (Huang and Liu, 2020). So far, small oligomers consisting of the aggregation-prone Aβ_42_ have been identified as most damaging, while Aβ_40_ is considered as rather neuroprotective, inhibiting Aβ_42_ aggregation (Vadukul et al., 2020; Jan et al., 2008). As a result, many *in vitro* studies use only pure Aβ_42_ oligomers, yet it is doubtful whether this approach emulates the *in vivo* tissue situation, where Aβ_42_ is mixed with Aβ isoforms of differing lengths (Deleanu et al., 2022). For instance, N-terminally truncated Aβ isoforms like Aβ_4-42_ form stable oligomeric aggregates and promote fibril formation whilst being similarly toxic as Aβ_42_ aggregates (Bouter et al., 2013). These N-terminally truncated isoforms and full-length Aβ species are expressed by various cells in the brain, including neurons, astroglia and oligodendrocytes (Sasmita et al., 2024; Hampel et al., 2021). In inflammatory conditions, such as in AD, the production of Aβ, and especially truncated isoforms, is even more enhanced, thereby aggravating disease progression (Meraz-Ríos et al., 2013; Beretta et al., 2024). Additionally, the inflamed state of microglia, astroglia and oligodendrocytes as well as associated cellular dysfunction fuel amyloid aggregation (Kenigsbuch et al., 2022; Alasmari et al., 2018). Moreover, Aβ_40_ is much more abundant in the brain compared to Aβ_42_, highlighting the strong probability of Aβ_42_ and Aβ_40_ co-aggregation as one of the earliest pathophysiological changes in the disease (Zoltowska et al., 2016). Various reports have indicated that the Aβ_42_:Aβ_40_ ratio determines toxicity, an important circumstance considering the development of new therapeutic antibodies only targeting pure Aβ_42_ oligomers (Kwak et al., 2020; Kuperstein et al., 2010; Chang and Chen, 2014; Sandberg et al., 2022).

So far, studies investigating the Aβ_42_:Aβ_40_ ratio mainly focused on physiochemical properties, and with evaluation of toxicity having been mostly restricted to neurons, toxicity induced by mixed Aβ oligomers may have been overlooked (Chang and Chen, 2014; Deleanu et al., 2022; Jan et al., 2008). Specifically, Aβ oligomers cause or exacerbate various detrimental processes, encompassing mitochondrial dysfunction, lipid dysregulation, (neuro-)inflammation, and blood–brain barrier (BBB) breakdown (Solis et al., 2020; Tarawneh, 2023; Hernandez-Zimbron et al., 2012; Singh, 2022; Zhao et al., 2023). Mitochondrial dysfunction, for instance, arises early in AD, resulting in decreased ATP production and rising levels of reactive oxygen species (Hernandez-Zimbron et al., 2012; Moreira et al., 2010). The associated release of mitochondrial cytochrome into the cytosol is an inducer of apoptosis, amplified by overexpression of cytochrome c (Chandra et al., 2002; Moreira et al., 2010). Glial cells like astroglia and microglia further contribute to neurodegeneration by shifting toward a pro-inflammatory state, elevating cytokine secretion and altering lipid metabolism (de Dios et al., 2023; Franciosi et al., 2005). In this context, lipid droplets have been central to recent research due to their dynamic involvement in cell metabolism (Ralhan et al., 2021; Zhao et al., 2023). Especially cholesterol and unsaturated lipids have been identified as essential contributors to AD, but their exact pathophysiological role remains elusive (Loving and Bruce, 2020). While unusual cholesterol accumulation is generally associated with AD, the impact of unsaturated and saturated lipids is more ambiguous (Loving and Bruce, 2020). For example, unsaturated lipids like anti-inflammatory *ω*-3 fatty acids contrast polyunsaturated lipids like prostaglandins, which sustain inflammation (Yin, 2023). Another still mostly unexplored function of particularly microglia is the formation of tunneling nanotubes (TNTs), which form bridges between distant cells, allowing for communication and organelle transfer (Wang and Gerdes, 2015). They have also been identified as a transportation system for Aβ, potentially explaining the spread of toxic peptides in the central nervous systems (Dilna et al., 2021; Wang and Gerdes, 2015). In addition, AD-related aberrant angiogenesis has been observed in *in vitro* studies, implying direct damaging effects on endothelial cells (Parodi-Rullán et al., 2020). In a tissue-context, the convergence of these single-cell effects leads to the breakdown of the BBB (Parodi-Rullán et al., 2020; Tarawneh, 2023; Solis et al., 2020). Despite the significant scientific interest in each of these individual mechanisms, they have mainly been studied *in vitro* using pure Aβ_42_ oligomers, which lack the physiological relevance of oligomers composed of varying A*β*_42_:Aβ_40_ ratios.

Quantitative *in vitro* assays are crucial for elucidating such mechanisms, however, obtaining comprehensive biochemical information is equally important. In this regard, confocal Raman microscopy has recently gained traction as a label-free technique, giving simultaneous insight into various biomolecular processes. Based on the inelastic scattering of light, Raman spectra provide specific chemical signatures stemming from the unique combination of bonds in molecules such as desoxyribonucleic acid (DNA), ribonucleic acid (RNA), lipids, or proteins (Jung et al., 2021; Pezzotti, 2021). Confocal Raman microscopy holds a distinct advantage over fluorescence microscopy due to the latter’s limited number of fluorescence channels and the requirement of a known target. By scanning cells or tissues, hyperspectral Raman data containing spatially resolved, unbiased biochemical information is obtained, which is especially valuable in elucidating poorly understood molecular changes. Additionally, its sensitivity to molecular conformation makes Raman spectroscopy a valuable tool for studying Aβ aggregation, which involves the conversion of *α*-helical secondary structure to β-sheets (Mallesh et al., 2023). Thus, both Raman imaging and spectroscopy hold great potential for analyzing neurodegeneration but are underutilized due to the complexity of the resulting spectral data, requiring specific expertise.

**Figure 1:**
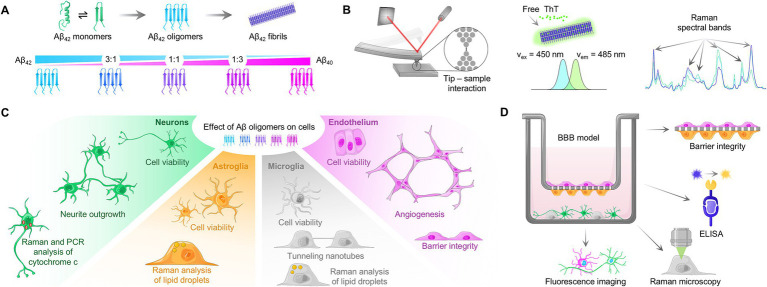
Study setup. **(A)** Schematic process of Aβ aggregation, involving conversion of α-helical secondary structure to β-sheets. By mixing varying ratios of Aβ_42_:Aβ_40_ and subsequent aggregation, mixed oligomers can be obtained. **(B)** Characterization of the amyloid aggregation process with atomic force microscopy (AFM), Thioflavin T assay and Raman spectroscopy. In AFM, interaction with sample matter leads to tip deflection, and thus movement of the laser spot on the detector, generating a topographic signal. Upon insertion of Thioflavin T in fibrillar structure, excitation and emission maxima are shifted, allowing for kinetic analysis. Changes in Raman spectral bands during Aβ aggregation can be measured using Raman spectroscopy. **(C)** Peptide solutions containing pure and mixed oligomers were used to study cell viability and functionality of neurons, astroglia, microglia, and endothelial cells. **(D)** Schematic overview of the human-based BBB model used in this study and the performed assays.

In this study, we combined the potential of confocal Raman microscopy and spectroscopy with various *in vitro* assays to comprehensively examine the influence of the Aβ_42_:Aβ_40_ ratio in oligomers on pathophysiological alterations in cells (Figure 1). State-of-the-art techniques enabled detailed physicochemical characterization, including the Thioflavin T (ThT) assay to study the impact of the Aβ_42_:Aβ_40_ ratio on aggregation kinetics, and atomic force microscopy (AFM) combined with Raman spectroscopy to distinguish between different types of Aβ aggregates. Furthermore, we examined the impact of pure and mixed oligomers on cell types critically affected by AD, specifically endothelial cells, neurons, astroglia, and microglia. By conducting an individual analysis for each cell type, we investigated effects on viability, functionality, and metabolism, thereby improving the understanding of Aβ oligomer toxicity. Of particular interest were neuronal dysfunction, differences in the composition of lipid droplets in astroglia and microglia, TNT formation, effects on endothelial barrier integrity, and angiogenesis. Lastly, we examined the effects of selected Aβ oligomers in a human *in vitro* model of the BBB, allowing for cellular interactions in a tissue microenvironment. Overall, our study enabled a detailed assessment of the effects of Aβ_42_:Aβ_40_ ratio in oligomers on multiple cell types of the brain by systematically unraveling molecular changes and functional impairment upon Aβ-induced toxicity.

## 2 Materials and methods

If not stated otherwise, chemicals and cell culture reagents were purchased from Merck KGaA (Darmstadt, Germany) and Thermo Fisher Scientific Inc. (Waltham, United States). Ultrapure water was purified with a PURELAB Flex 2 system (Veolia Water Technologies Deutschland GmbH, Celle, Germany).

### 2.1 Aβ preparation

Aβ_42_ or Aβ_40_ peptide (Kaneka Eurogentec S.A., Seraing, Belgium) was dissolved in hexafluoroisopropanol (HFIP, Th. Geyer GmbH & Co. KG, Renningen, Germany) and incubated for 2 h at room temperature. Then, peptides were optionally mixed to ratios of Aβ_42_:Aβ_40_ of 3:1 (Aβ_3:1_), 1:1 (Aβ_1:1_), and 1:3 (Aβ_1:3_) and aliquoted in Eppendorf tubes. HFIP was evaporated overnight in a vacuum, and the dry peptide films were stored at −70°C until use.

### 2.2 Thioflavin T fluorescence assays

Dried peptide films (Pure Aβ_42_, Aβ_3:1_, Aβ_1:1_, Aβ_1:3_ and pure Aβ_40_) were dissolved in 50 mM sodium hydroxide to a 45.5 μM solution and bath sonicated for 5 min. Next, peptide solutions were diluted to 20 μM in black 96-well plates using 60 mM HCl and supplemented with 20 μM Thioflavin T (ThT, Biomol GmbH, Hamburg, Germany). For the vehicle control, the same procedure was repeated but without peptide. The assay was performed using a TECAN Spark plate reader and a humidity cassette (TECAN Group AG, Männedorf, Switzerland) for 6 h at 37°C with excitation at 448 ± 7 nm and emission at 485 ± 20 nm, measuring every 5 min. For data analysis of each experimental run, the intensity of the vehicle control was subtracted from the intensities recorded for the different peptide ratios.

### 2.3 Aβ aggregation

Peptide aggregation protocols were adapted from the protocol by Stine et al. (2011). Briefly, monomeric Aβ_42_ was prepared by dissolving dried peptide film in cold 0.02% ammonia for 10 min on ice and adding ice-cold sterile ultrapure water to a final concentration of 100 μM. Oligomers of Aβ_42_ (OAβ_42_), Aβ_1:1_ (OAβ_1:1_), and Aβ_3:1_ (OAβ_3:1_) were obtained by initially dissolving peptide in 0.02% ammonia, followed by sterile ultrapure water and incubation at 4°C overnight. Fibrils of Aβ_42_ (FAβ_42_), oligomers of Aβ_40_ (OAβ_40_), and Aβ_1:3_ (OAβ_1:3_) were obtained by adding sterile 10 mM HCl and incubating at 37°C overnight instead. All peptide solutions and aggregates were freshly prepared for each experiment.

### 2.4 Atomic force microscopy

Atomic force microscopy (AFM) was performed on a JPK Nanowizard III (Bruker Corporation, Billerica, United States) using ACTA tips (APPNano, Mountain View, United States) with the following properties: tip-radius < 10 nm, spring constant 37 N/m, frequency 300 kHz, 125 μm length, 30 μm width, 4 μm thickness, and an aluminum cantilever coating. Samples were prepared as follows: cleaned mica sheets were glued to microscopy slides using epoxy glue and dried overnight. Then, they were transferred to a laminar-air-flow bench and washed using 300 μL of 96% ethanol (VWR International GmbH, Darmstadt, Germany), and left to dry inside the bench. Mica was cleaved using tape strips for 2–4× until a smooth surface free from cracks was visible. Next, freshly prepared peptide solutions were diluted using ultrapure water to a concentration of 20 μM (monomers, oligomers) or 5 μM (fibrils). Samples were immediately spotted on the mica and incubated for 10 min. Using 100 μL of filtered (0.22 μm) ultrapure water, excess sample solution was washed off. Afterward, samples were placed in a vacuum and stored for a maximum of 16 h before AFM imaging.

### 2.5 Drop-coating deposition Raman spectroscopy (DCDRS), spectral processing, cross-correlation analysis and peak ratio analysis

Ten microliters drops of peptide solution were placed on calcium fluoride dishes (Korth Kristalle GmbH, Altenholz, Germany) and dried for 30 min in a fume hood. Raman spectra at 30 randomly chosen positions in the middle of the dried drop were acquired with a confocal Raman microscope alpha300R (WITec GmbH, Ulm, Germany) equipped with a 50x objective (NA 0.8) and a 532 m laser set to a power of 20 mW in front of the objective. Integration time was set to 2–8 s. Background and cosmic rays were removed using the ProjectFour software (WITec GmbH, Ulm, Germany). Preprocessed spectra were then imported to Matlab (The Mathworks Inc., Natick, United States). Using the spectra of Aβ_42_ monomers, oligomers, and fibrils, a cross-correlation analysis was performed to investigate which Raman peaks are subject to sequential change (Lasch and Noda, 2019). With this knowledge, specific peak ratios (1,240 cm^−1^/1307 cm^−1^, 1,671 cm^−1^/1447 cm^−1^, 1,671 cm^−1^/1555 cm^−1^, 1,671 cm^−1^/1609 cm^−1^, and 2,935 cm^−1^/2850 cm^−1^) were chosen for peak ratio analysis according to the assignments in Supplementary Table S1.

### 2.6 General cell culture

Human SH-SY5Y neuroblastoma cells (Cell lines services, Eppelheim, Germany) were cultured in DMEM with 10% fetal calf serum (FCS) and used up for up to 8 passages (maximum passage 12). Human CCF-STTG1 astroglia (ATCC, Manassas, United States) were cultured in RPMI with 10% FCS and used up for up to 10 passages (maximum passage 12). Human HMC3 microglia (ATCC, Manassas, USA) were cultured in a-MEM with 10% FCS and used for up to 10 passages (maximum passage 12). The human cerebral microvascular endothelial cell line hCMEC/D3 (Cedarlane Labs, Burlington, United States) was cultured on collagen-coated flasks (10 μg/cm^2^) in EGM-2 medium (Promocell, Heidelberg, Germany) used for up to 6 passages (maximum passage 34). Cells were passaged by trypsinization every 7 days. hCMEC/D3 endothelial cells were used for assays at 30,000 cells/cm^2^, differentiated SH-SY5Y at 45,000 cell/cm^2^, HMC3 microglia and CCF-STGG1 astroglia at 24,000 cells/cm^2^, if not stated otherwise.

### 2.7 Immunofluorescence staining and imaging

All fluorescence staining followed a standardized procedure: after fixation, cells were washed 3× in PBS + 0.05% Tween 20 (PBS-T) for 5 min and permeabilized with 0.2% Triton-X for 5 min. Samples were washed again 3x in PBS-T for 5 min. For antibody staining, samples were first blocked in 5% goat serum and 5% BSA for 20 min. Then, without washing, primary antibody (1:1000 *β*-III tubulin mouse-anti-human, Thermo Fisher Scientific, Waltham, United States) in blocking buffer (5% bovine serum albumin +5% goat serum in PBS) was added and incubated under gentle shaking at 4°C overnight. Samples were washed 3× in PBS-T for 5 min, and secondary antibody in PBS (1:400 Alexa Fluor 633 goat-anti-mouse) was added and incubated under gentle shaking for 1 h at room temperature. Subsequently, samples were washed 3x with PBS-T for 5 min. For actin staining, samples were incubated after permeabilization and washing with Alexa Fluor 488 Phalloidin or Alexa Fluor 555 Phalloidin under shaking conditions for 1 h at room temperature. Then, samples were washed 3× in PBS-T for 5 min. After the respective staining (antibody or phalloidin or a combination) and washing, samples were counterstained with DAPI (1:100) in PBS under shaking for 5 min at room temperature. After washing 2× in PBS-T and 1x in ultrapure water for 5 min each, samples were mounted in FluorSave (Merck KGaA, Darmstadt, Germany) and dried overnight. Samples were stored at 4°C. Imaging was performed using an inverted confocal laser scanning microscope equipped with 10× (NA 0.45), 20× (NA 0.8), and 40× oil immersion (NA 1.4) objectives (Zeiss, Oberkochen, Germany).

### 2.8 SH-SY5Y cell differentiation

SH-SY5Y neuroblastoma cell differentiation protocols were adapted from existing literature using retinoic acid and brain-derived neurotrophic factor (BDNF), which generates an excitatory neuronal population with glutamatergic and cholinergic markers (Encinas et al., 2000; Dravid et al., 2021; Shipley et al., 2016; Targett et al., 2024). Briefly, SH-SY5Y cells were seeded in T-75 flasks at 10,000 cells/cm^2^ in DMEM supplemented with 10% FCS and left to adhere overnight. On day 1 post seeding, medium was changed to DMEM supplemented with 2.5% FCS, 2 mM glutamine and 10 μM retinoic acid. On day 4 post seeding, medium was changed to Neurobasal™ medium enriched with 50 ng/mL BDNF, 1x B-27(TM) supplement, and 10 μM retinoic acid. On day 7 post seeding, cells were gently passaged, counted, and seeded for experimental use. To verify differentiation, fluorescence staining of *β*-III tubulin was performed according to the protocol above (Supplementary Figure S2).

### 2.9 Viability assay

The respective cells were seeded in flat-bottom 96-well plates and incubated overnight. The next day, cells were treated with pure and mixed oligomers at 10, 5, 2.5, 1.25, and 0.6125 μM, medium (control), or PBS. Treatment with 100 ng/mL LPS + 20 ng/mL IFN-y (Immunotools GmbH, Friesoythe, Germany) was included for HMC3 microglia. After 24 h, medium was discarded, and fresh medium supplemented with 1 mg/mL 3-(4,5-dimethylthiazol-2-yl)-2,5-diphenyltetrazolium bromide (MTT) reagent was added and incubated for 4 h at 37°C. Then, medium was discarded again, 100 μL of DMSO was added per well, and the plate was placed on a lab shaker for 15 min. After ensuring the formazan crystals had dissolved, the absorption was measured at 570 nm in a TECAN Spark plate reader (TECAN Group AG, Männedorf, Switzerland). For data evaluation, viability was normalized to the control.

### 2.10 Neurite outgrowth assay

To further verify the differentiation of SH-SY5Y cells, neurite outgrowth was compared between SH-SY5Y neuroblastoma cells and differentiated SH-SY5Y neurons. Briefly, cells were seeded in a 24-well plate left to settle for 24 h. The next day, phase contrast images were acquired using an inverted Leica DMi8 microscope (LEICA microsystems, Wetzlar, Germany) equipped with a 20x objective. Images were then imported to Fiji, converted to 8-bit images and neurite length was measured using the NeuronJ plugin (Meijering et al., 2004; Schindelin et al., 2012). For the investigation of the toxicity of pure and mixed Aβ oligomers, differentiated cells were seeded in 24-well plates in Neurobasal™ medium enriched with 1x B-27(TM) supplement and treated with 10 μM of the respective peptide solution or medium (control) for 24 h. Neurite length was measured as described above.

### 2.11 PCR analysis of cytochrome c messenger RNA levels

Briefly, differentiated SH-SY5Y cells were seeded in a 24-well plate and incubated for 24 h, followed by 24 h treatment with 10 μM OAβ_42_, OAβ_1:3_, or medium. Cells were lyzed via the addition of 350 μL TRIzol™ per well, and total RNA was isolated using the Direct-zol RNA MiniPrep Plus Kit (Zymo Research Europe GmbH, Freiburg, Germany) according to the manufacturer; concentration as well as purity were validated photometrically in a TECAN Spark plate reader using the NanoQuant Plate (TECAN Group AG, Männedorf, Switzerland). First-strand synthesis was performed with the Maxima H Minus cDNA Synthesis Master Mix Kit (Thermo Fisher Scientific, Waltham, MA, United States), implementing 250 ng total RNA and following the protocol provided by the manufacturer. Afterward, complementary desoxyribonucleic acid (cDNA) concentration was measured as mentioned above. Cytochrome c (CYC1) messenger RNA (mRNA) level were analyzed via the StepOnePlus Real-Time PCR System (Applied Biosystem, Waltham, MA, United States) using the SYBR Green PowerTrack Master Mix (Thermo Fisher Scientific, Waltham, MA, United States) and 10 ng cDNA. Resulting *C*t values were evaluated according to the 2^−ΔΔCt^ method employing glyceraldehyde-3-phosphate dehydrogenase (GAPDH) as a housekeeping gene. Data were subsequently normalized to the untreated control (Livak and Schmittgen, 2001). The following primer pairs (5′–3′ orientation) were used for amplification: GAPDH *forward*: CGGGAAGCTTGTCATCAATGG, GAPDH *reverse*: GGCAGTGATGGCATGGACTG, CYC1 *forward*: CGGAGGTGGAGGTTCAAGAC, and CYC1 *reverse*: TAGAGACCTTCCCGCAGTGA. Primer efficiency was routinely validated before the experiment.

### 2.12 Confocal Raman microscopy of cells

The respective cells were seeded on calcium fluoride dishes (Korth Kristalle GmbH, Altenholz, Germany) and incubated overnight. The next day, cells were treated with peptide solutions at 10 μM or medium (control) and incubated for 24 h. Then, cells were fixed in 4% formaldehyde (in PBS) for 10 min, and subsequently washed 3x in PBS. Using a confocal Raman microscope (WITec GmbH, Ulm, Germany) equipped with a 532 nm laser set to a power of 37 mW and a 63× water dipping objective, Raman scans were acquired with an integration time of 0.2 s and a spatial resolution of 500 nm. Background of scans was subtracted using the ProjectFOUR software (WITec GmbH, Ulm, Germany). Scans were then imported to Matlab (The Mathworks Inc., Natick, United States) to remove cosmic rays, smooth the data (Savitzky–Golay filter, window size 9, order 3), and normalized using the Stand Normal Variate method. Next, data was analyzed using the endmember analysis algorithm N-FINDR (Winter, 1999). Briefly, the number of endmembers in the hyperspectral data set was estimated using the noise-whitened Harsanyi-Farrand-Chang (NWHFC) method. End-member spectra were identified by the N-FINDR algorithm and used to calculate end-member abundance maps. Individual abundance maps were overlayed using Fiji. Spectra were plotted, and peak ratio analysis (refer to detailed peak assignment in Supplementary Table S1) was conducted for cytochrome c and/or lipids. Raman imaging was divided into a pre-study to assess feasibility and a subsequent study which encompassed Raman spectra from 3 to 8 cells in total.

### 2.13 Tunneling nanotube quantification

Tunneling nanotube (TNT) measurement was adapted from existing protocols (Chakraborty et al., 2023; Kretschmer et al., 2019). Briefly, HMC3 microglial cells were seeded on high-precision glass coverslips (170 ± 5 μm) and incubated overnight. The next day, cells were treated with 10 μM peptide solution or medium (control) and incubated for 24 h. HMC3 cells were then fixed using a two-step fixation protocol: 15 min with 2% formaldehyde and 0.05% glutaraldehyde (Carl Roth GmbH + Co. KG, Karlsruhe, Germany) in PBS, followed by 15 min in 4% formaldehyde in PBS. Subsequently, phalloidin and DAPI staining were performed according to the protocol above, and glass slips were mounted in FluorSave (Merck KGaA, Darmstadt, Germany). Z-stacks were acquired using an inverted confocal laser scanning microscope (LSM900, Carl Zeiss AG, Oberkochen, Germany) using a 20× objective and 0.5 μm step size. For each n and treatment group, 3 z-stacks were acquired at random locations in the sample. Stack scans were then imported to ICY Bioimage analysis software, and TNTs were counted using the TNT annotation tool. A TNT was only counted if the cellular structure hovered above ground level (assessed via z-stack-based spatial resolution of the acquired fluorescence images), if its length exceeded 5 μm, and if its thickness was below 1 μm (Thayanithy et al., 2017; Matejka and Reindl, 2019).

### 2.14 Transepithelial electrical resistance (TEER) measurement

hCMEC/D3 endothelial cells were seeded at 40,000 cells/cm^2^ on 24-well PET 0.4 μm pore inserts (Brand GmbH + Co KG, Wertheim, Germany) in EGM-2 medium (Promocell, Heidelberg, Germany) without VEGF (vascular endothelial growth factor), supplemented with 50 μg/mL ascorbic acid and 1.4 μM hydrocortisone (Caesar & Loretz GmbH, Hilden, Germany). TEER was measured daily using an the Millicell^®^ ERS-2 (Merck KGaA, Darmstadt Germany) equipped with an EVOM2 electrode (World precision instruments, Sarasota, United States). Plateau TEER was reached on day 3 post seeding, at which point cells were treated with peptide solutions at 10 μM or medium (control) and incubated for 24 h, followed by a final TEER measurement. This experiment was performed in quadruplicate.

### 2.15 Angiogenesis assay

The angiogenesis assay was adapted from existing literature (Thomas et al., 2017; Faulkner et al., 2014). Briefly, hCMEC/D3 endothelial cells were starved in DMEM without FCS overnight. Geltrex™ (Thermo Fisher Scientific, Waltham, United States) was thawed at 4°C on ice overnight. The next day, a flat-bottom 96-well plate and 10 μL pipette tips were pre-cooled at −20°C for 30 min, and 2.5 μL of Geltrex™ was pipetted into each well. The plate was then placed at 37°C for 30 min. Meanwhile, hCMEC/D3 cells were passaged and counted. After incubation of Geltrex™ in the plate, hCMEC/D3 cells were seeded in EGM-2 basal medium (Promocell, Heidelberg, Germany). After 4 h, cells were treated with peptide solutions at 10 μM, medium (control), or vascular endothelial growth factor (VEGF, positive control). After 20 h, cells were fixed in 4% formaldehyde (in PBS) for 10 min. Phalloidin staining, followed by DAPI staining, was performed according to the protocol above. Fluorescence images for quantitative analysis were acquired using an inverted Leica DMi8 microscope (LEICA microsystems, Wetzlar, Germany) equipped with a 5× objective. Angiogenesis was assessed using the REAVER script in Matlab (The Mathworks Inc., Natick, United States) with standard settings except for a gray threshold of 0.05, averaging filter size of 500, minimum connected component area of 100, and vessel thickness threshold of 3 (Corliss et al., 2020).

### 2.16 Blood–brain barrier model cultivation

On day 1, 40,000 CCF-STTG1 astroglia were seeded in RPMI +10% FCS on collagen-coated (0.9 mg/mL collagen, 15 μL) basal sides of 24-well inserts (Falcon^®^ cell culture inserts, 0.4 μm pore size, Corning Inc., Corning, United States) and left to attach overnight. On day 2, hCMEC/D3 endothelial cells (50,000 cells) were seeded on the apical side of the insert membrane in EGM-2. HMC3 microglia (5,000 cells) and differentiated SH-SY5Y neurons (45,000 cells) were seeded in the basal compartment on collagen-coated coverslips (0.1 mg/mL, 900 μL, Corning Inc., Corning, United States) in a-MEM + 10% FCS. On day 3, the medium was exchanged for a-MEM + 2% FCS. After initial TEER measurement on day 4, the basolateral compartment was supplemented with 10 μm Aβ_42_, Aβ_1:3_, 100 ng/mL LPS + 20 ng/mL IFN-*γ* or medium/PBS (control), respectively, and cells were cultivated for additional 24 h.

### 2.17 Blood–brain barrier model analysis

TEER was measured on day 5 after 24 h incubation with the respective treatment. TEER measurement was performed in quadruplicate. The medium of the basal compartment was collected for enzyme-linked-immunosorbent-assay (ELISA). Briefly, 800 μL of medium was collected per well and centrifuged at 14,000 rpm for 15 min at 4°C to remove cell debris. The supernatant was frozen at −70°C and thawed 1 h prior to analysis. ELISAs of IL-6 and IL-8 were performed according to the manufacturer’s instructions (Thermo Fisher Scientific, Waltham, United States). For fluorescence imaging and Raman microscopy, cells were fixed according to the TNT cell fixation protocol above or with 4% formaldehyde (in PBS) for 10 min, respectively. Raman microscopy was performed in duplicate. Antibody and phalloidin staining were conducted according to the protocol above on cells fixed with the TNT fixation protocol.

### 2.18 Statistical analysis

Results are shown as mean values ± standard deviation indicated by error bars. Each experiment included technical triplicates and was performed in triplicate if not stated otherwise. Statistical analysis was performed for the following experiments using the specified test. ANOVA followed by Dunnett *post hoc* test: DCDRS peak ratio analysis, viability assay, neurite outgrowth assay, PCR analysis of cytochrome c mRNA levels, tunneling nanotube quantification, angiogenesis assay, IL-6 and IL-8 secretion in the BBB model; student’s paired *t*-test: TEER measurements on endothelial cell monocultures, TEER measurements in the BBB model; two sample *t*-test: neurite outgrowth of differentiated neurons. Statistical significance is indicated by *p < 0.05, **p < 0.01, and ***p < 0.001 in the figures. If a statistical test was performed, but no significance is indicated, the test was non-significant.

## 3 Results

### 3.1 Characterization of the Aβ aggregation process and individual Aβ aggregates

**Figure 2:**
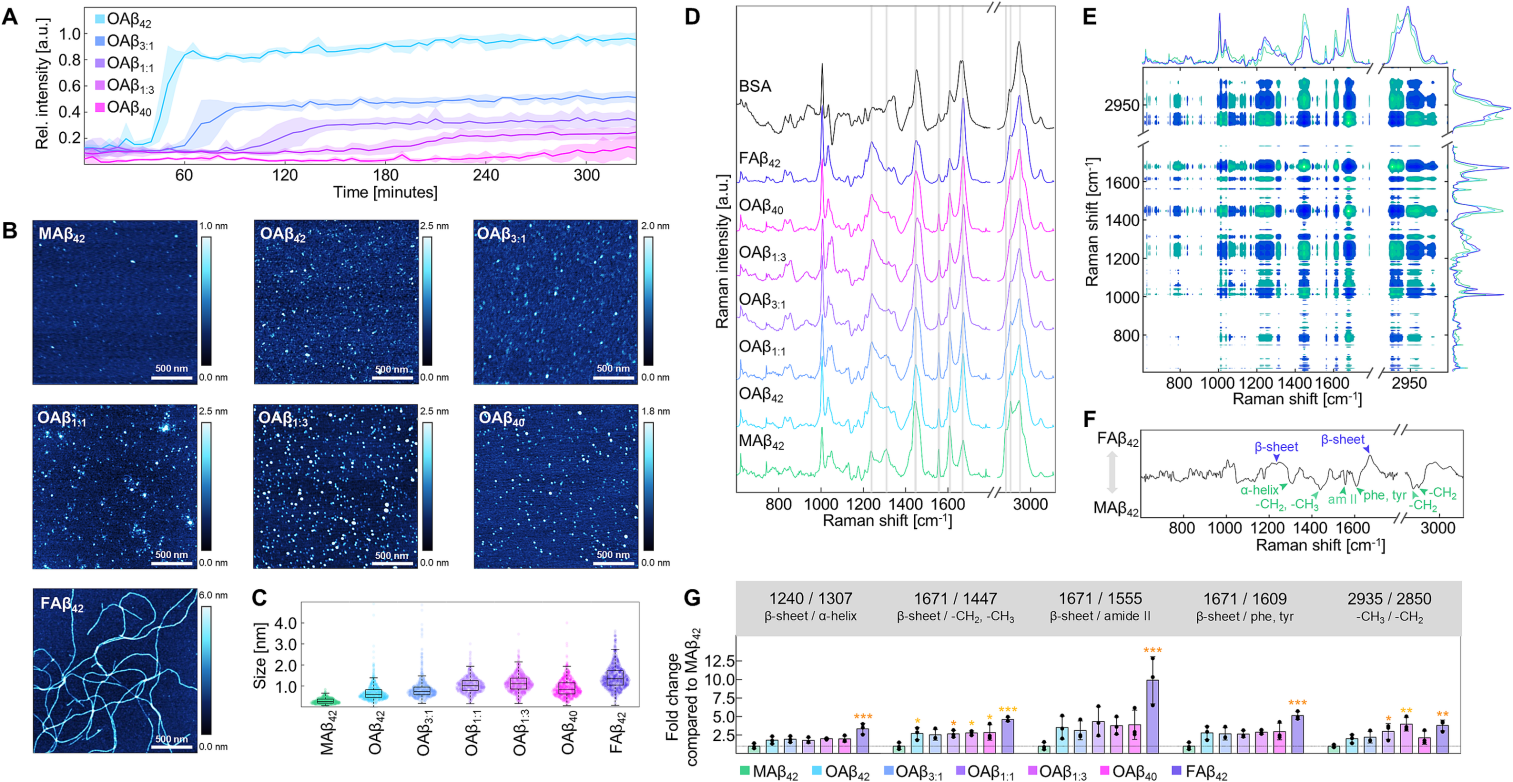
Characterization of the Aβ aggregation process and aggregates of different Aβ_42_:Aβ_40_ ratios. **(A)** ThT aggregation profiles for varying ratios of Aβ_42_:Aβ_40_ at 20 μM (*n* = 3). **(B)** Exemplary AFM topographies for monomers, oligomers, and fibrils of Aβ. Topographies were acquired from dried AFM slides covered with 20 μM (monomers, oligomers) or 5 μM (fibrils) peptide solutions. **(C)** Size measurement MAβ_42_, OAβ_42_, OAβ_3:1_, OAβ_1:1_, OAβ_1:3_, OAβ_40_, and FAβ_42_ using AFM (*n* = 3). **(D)** Raman spectra of MAβ_42_, OAβ_42_, OAβ_3:1_, OAβ_1:1_, OAβ_1:3_, OAβ_40_, FAβ_42_, and bovine serum albumin, which contains mainly α-helical secondary structure. Raman peaks differing between the aggregate types are indicated by gray bands (*n* = 3). Raman spectra were acquired from dried 100 μM peptide films (drop-coating deposition Raman spectroscopy). **(E)** Cross-correlation analysis of Raman spectra derived from MAβ_42_, OAβ_42_, and FAβ_42_ (*n* = 3). Note the diagonal axis of symmetry indicating self-correlated peaks. Vertical and horizontal lines reveal correlation of one peak with all other peaks. Positive correlation is observable if both examined peaks have the same color in the plot, whereas inverse correlated peaks possess opposite colors. **(F)** Synchronous correlation spectrum at 1670 cm^−1^, a β-sheet peak. Maxima are positively correlated, whilst minima are inversely correlated with β-sheet peak intensity. Notable maxima and minima are indicated. **(G)** Peak ratio analysis of all examined Aβ aggregates (*n* = 3) showing the fold change of the respective ratio normalized to MAβ. Statistical significance is indicated compared to MAβ. *p < 0.05, **p < 0.01, ***p < 0.001, am II, amide II; phe, phenylalanine; tyr, tyrosine.

We first investigated the aggregation kinetics of pure and mixed monomeric Aβ_42_ and Aβ_40_ in differing ratios by performing a Thioflavin T (ThT) assay, revealing gradual differences in aggregation curves (Figure 2A). While the Aβ_40_ profile did not reach a plateau in the examined time interval, Aβ_42_ attained a steady state at 0.87 ± 0.07 a.u. after 65 min. The mixed amyloid curves were distributed between the profiles of pure Aβ_42_ and Aβ_40_, differing in steepness of the sigmoid profile and height of the fluorescence plateau. Specifically, profiles of Aβ_42_:Aβ_40_ mixtures with a ratio of 3:1 (Aβ_3:1_), equal mixtures of both species (Aβ_1:1_) and the combination of both species in a 1:3 ratio (Aβ_1:3_) reached their plateau after 110 min at 0.45 ± 0.02 a.u, 155 min at 0.31 ± 0.04 a.u., and after 225 min at 0.22 ± 0.05 a.u, respectively.

Subsequently, we prepared oligomers of pure and mixed Aβ_42_ and Aβ_40_, (OAβ_42_, OAβ_3:1_, OAβ_1:1_, OAβ_1:3_, OAβ_40_) as well as monomers (MAβ_42_) and fibrils of Aβ_42_ (FAβ_42_). Characterization of these aggregate types using atomic force microscopy (AFM) uncovered circular structures for Aβ monomers and oligomers and elongated structures for fibrils (Figure 2B). By measuring aggregate height in each AFM topography, serial increases in size were observable from MAβ_42_, to OAβ_42_ and FAβ_42_, sized 0.29 ± 0.08 nm, 0.69 ± 0.22 nm, and 1.43 ± 0.10 nm, respectively (Figure 2C). OAβ_3:1_, OAβ_1:1_, OAβ_1:3_, and OAβ_40_ were sized 0.87 ± 0.22 nm, 1.04 ± 0.06 nm, 1.14 ± 0.11 nm, and 0.92 ± 0.03 nm, respectively.

Raman spectroscopy revealed changes in Raman peaks associated with *α*-helical (1,307 cm^−1^) and β-sheeted (1,240 and 1,671 cm^−1^) secondary structure, amide backbone (1,555 cm^−1^), phenylalanine and tyrosine sidechains (1,607–1,615 cm^−1^), as well as aliphatic side chains with -CH_2_,-CH_3_ deformation at 1,447 cm^−1^, -CH_2_ asymmetric stretching at 2,850 cm^−1^, -CH_2_ symmetric stretching at 2,885 cm^−1^, and symmetric -CH_3_ stretching at 2,935 cm^−1^ (Figure 2D) (Fonseca et al., 2019; Rygula et al., 2013; Mensch et al., 2017; Movasaghi et al., 2007; Kuhar et al., 2021; Jamieson et al., 2018). Next, we employed cross-correlation analysis of Raman spectra of Aβ_42_ monomers, oligomers, and fibrils. The synchronous spectrum depicted in Figure 2E represents a heatmap of the cross-correlation of all Raman shifts; the symmetry line stems from self-correlation. Thus, the correlation of one specific peak with all other peaks can be identified by examining the synchronous spectrum horizontally or vertically. Figure 2F shows the correlation spectrum at 1671 cm^−1^, a β-sheet peak, which is positively correlated with another β-sheet peak and negatively correlated with peaks of α-helices, aliphatic side chains, and amide backbone. Using ratios of inversely correlated peaks or peak ratios with constant denominators (2,935 cm^−1^), steady trends depending on aggregate type were found for each peak ratio (Figure 2G). However, differentiation of pure and mixed oligomers was impossible with this analysis.

### 3.2 Effect of pure and mixed Aβ oligomers on cell viability, function, and metabolism

**Figure 3:**
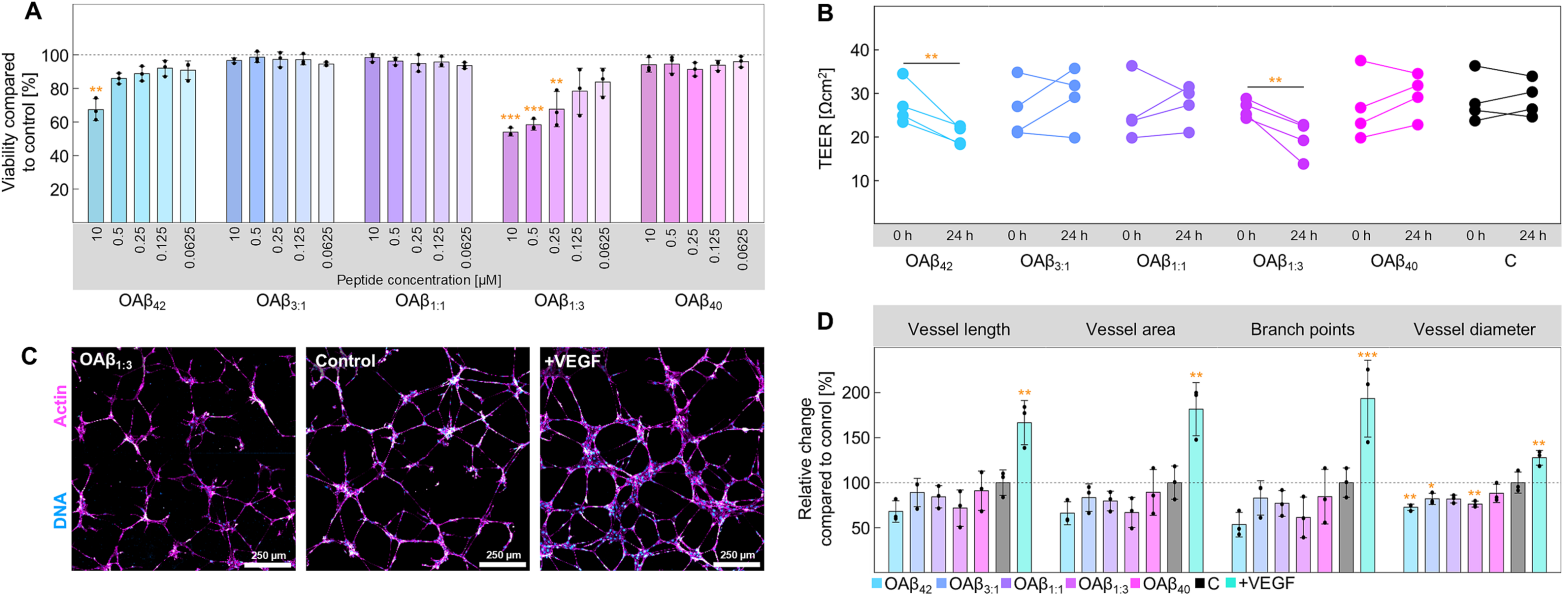
Cell viability and functionality of endothelial cells. **(A)** Viability of endothelial cells after treatment with varying concentrations of OAβ_42_, OAβ_3:1_, OAβ_1:1_, OAβ_1:3_, or OAβ_40_ (*n* = 3). Viability was normalized to the control (medium). Statistical significance is indicated compared to the control. **(B)** Results of TEER measurement before and after 24 h exposure to 10 μM OAβ_42_, OAβ_3:1_, OAβ_1:1_, OAβ_1:3_, OAβ_40_, or medium (control) (*n* = 4). Statistical significance is indicated compared to the 0 h timepoint. **(C)** Exemplary staining of DNA (blue) and actin (pink) after treatment with 10 μM OAβ_1:3_, medium (control), or VEGF (positive control). **(D)** Results of angiogenesis assay after treatment with 10 μM OAβ_42_, OAβ_3:1_, OAβ_1:1_, OAβ_1:3_, OAβ_40_, or medium (control) (*n* = 3) showing the relative change of the respective angiogenesis parameter (vessel length, vessel area, branch points or vessel diameter) compared to the control. Statistical significance is indicated compared to the control. *p < 0.05, **p < 0.01, ***p < 0.001, C, control.

Next, we systematically examined the effect of pure and mixed Aβ oligomers on four cell types – endothelial cells, neurons, astroglia, and microglia – to examine differential effects on cell viability, functionality, and metabolism. Endothelial cells form the essential base of the blood–brain barrier, constituting a tight cell monolayer. Whilst OAβ_3:1_, OAβ_1:1_, and OAβ_40_ did not affect cell viability, OAβ_42_ and OAβ_1:3_ significantly decreased viability in a concentration-dependent manner, to 67.30 ± 6.53% and 53.96 ± 2.36% at 10 μM, respectively (Figure 3A). Examination of barrier integrity via transepithelial electrical resistance (TEER) measurements exposed a significant reduction of this parameter in endothelial monolayers treated with OAβ_42_ (from 27.45 ± 4.93 Ωcm^2^ to 20.33 ± 2.18 Ωcm^2^) and OAβ_1:3_ (from 26.40 ± 2.04 Ωcm^2^ to 19.58 ± 4.18 Ωcm^2^) (Figure 3B). Consistently, the assessment of angiogenesis, encompassing vessel length, area, diameter, and branch points further manifested the indications of toxic effects predominantly induced by OAβ_42_ and OAβ_1:3_ (Figures 3C,D and Supplementary Figure S1).

**Figure 4:**
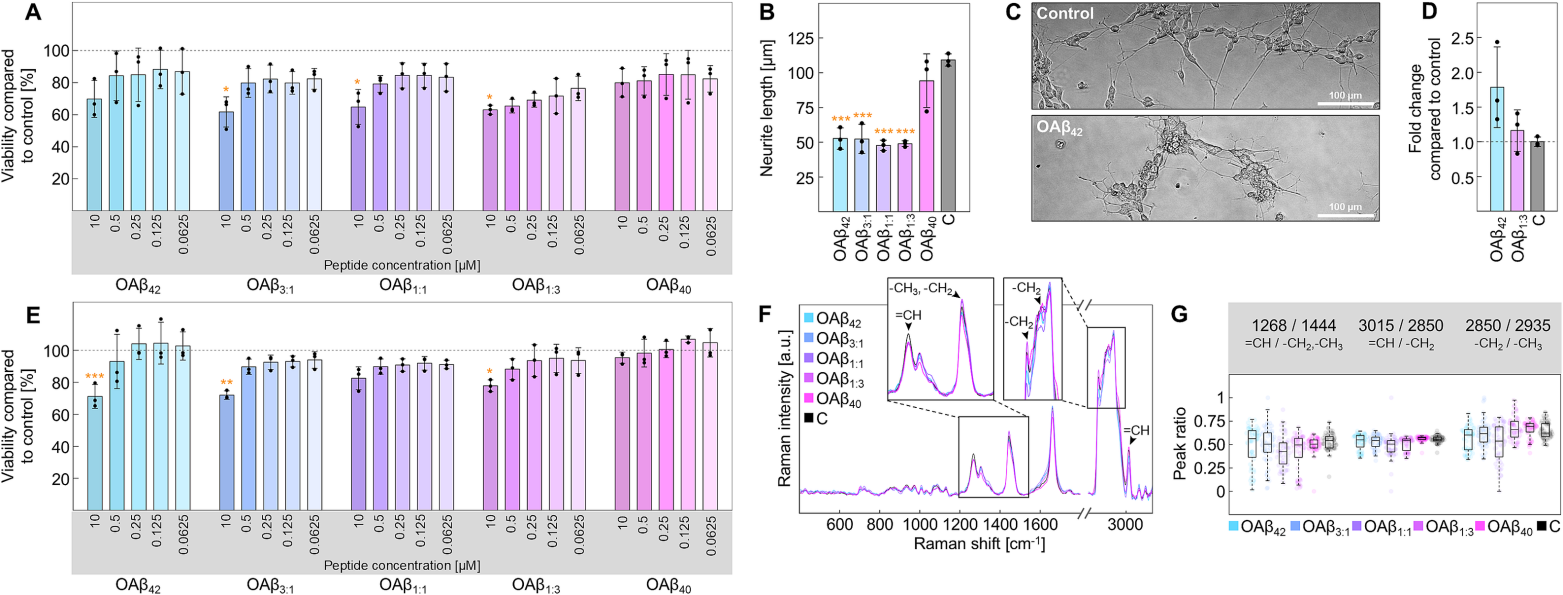
Cell viability and functionality of neurons and astroglia. **(A)** Viability of neurons after treatment with varying concentrations of OAβ_42_, OAβ_3:1_, OAβ_1:1_, OAβ_1:3_, or OAβ_40_ (*n* = 3). Viability was normalized to the control (medium). Statistical significance is indicated compared to the control. **(B)** Results of the neurite outgrowth assay after treatment for with 10 μM OAβ_42_, OAβ_3:1_, OAβ_1:1_, OAβ_1:3_, or OAβ_40_ (*n* = 3). Viability was normalized to the control (medium). Statistical significance is indicated compared to the control. **(C)** Exemplary phase contrast images of neurons in the neurite outgrowth assay after incubation. **(D)** Analysis of cytochrome c mRNA levels in neurons after exposure to 10 μM OAβ_42_, OAβ_1:3_, or medium (control) (*n* = 3), showing the fold change compared to the control. **(E)** Viability of astroglia after treatment with varying concentrations of OAβ_42_, OAβ_3:1_, OAβ_1:1_, OAβ_1:3_, OAβ_40_, or medium (control) (*n* = 3). Statistical significance is indicated compared to the control. **(F)** Raman spectra of lipid droplets of astroglia after treatment with 10 μM oligomers or medium (control). The zoomed sections and arrowheads mark typical lipid peaks. **(G)** Peak ratio analysis for peaks of interest. *p < 0.05, **p < 0.01, ***p < 0.001, C, control.

In the case of differentiated neurons (Supplementary Figure S2), the viability assay depicts concentration-dependent toxicity of OAβ_42_ and mixed oligomers (Figure 4A). Specifically, OAβ_42_, OAβ_3:1_, OAβ_1:1_, and OAβ_1:3_ at a concentration of 10 μM reduced cell viability to 69.70 ± 11.53%, 61.70 ± 9.41%, 64.77 ± 10.88%, and 63.03 ± 2.81%, respectively. OAβ_40_ exposure only led to a reduction of viability to 79.86 ± 8.88%. Further, results of the neurite outgrowth assay exposed an inhibited growth of these protrusions after incubation with mixed oligomers, approximating the effect of OAβ_42_ (Figures 4B,C and Supplementary Figure S3). We also evaluated the effect of OAβ_42_ and the mixed oligomer OAβ_1:3_, containing a small proportion of Aβ_42_, on cytochrome c mRNA levels, revealing a 1.16 ± 0.30-fold and 1.78 ± 0.58-fold increase, respectively (Figure 4D). Further, Raman analysis of neurons treated with Aβ oligomers also revealed a trend toward increasing intensities of cytochrome c at 750, 1,129, and 1,585 cm^−1^ from OAβ_40_, over mixed oligomers, to OAβ_42_ (Supplementary Figure S4; Brazhe et al., 2012).

Concerning astroglia, moderate cytotoxicity was registered for OAβ_42_, OAβ_3:1_, OAβ_1:1_, and OAβ_1:3_ at a concentration of 10 μM (71.17 ± 7.44%, 71.96 ± 2.64%, 82.47 ± 7.15%, and 77.83 ± 3.68%), with OAβ_1:1_ leading to the weakest effect, whereas viability was not affected by exposition to OAβ_40_ (Figure 4E). Further analysis of lipid droplets via confocal Raman microscopy did not reveal differences in lipid droplet distribution (Supplementary Figure S5), however, slight alterations in the corresponding Raman spectra were observed (Figure 4F). Employing peak ratio analysis, we found a lowered ratio of unsaturated to saturated lipids for cells treated with either mixed oligomers or OAβ_42_, but no trend in alterations in lipid chain length, as shown in Figure 4G.

**Figure 5:**
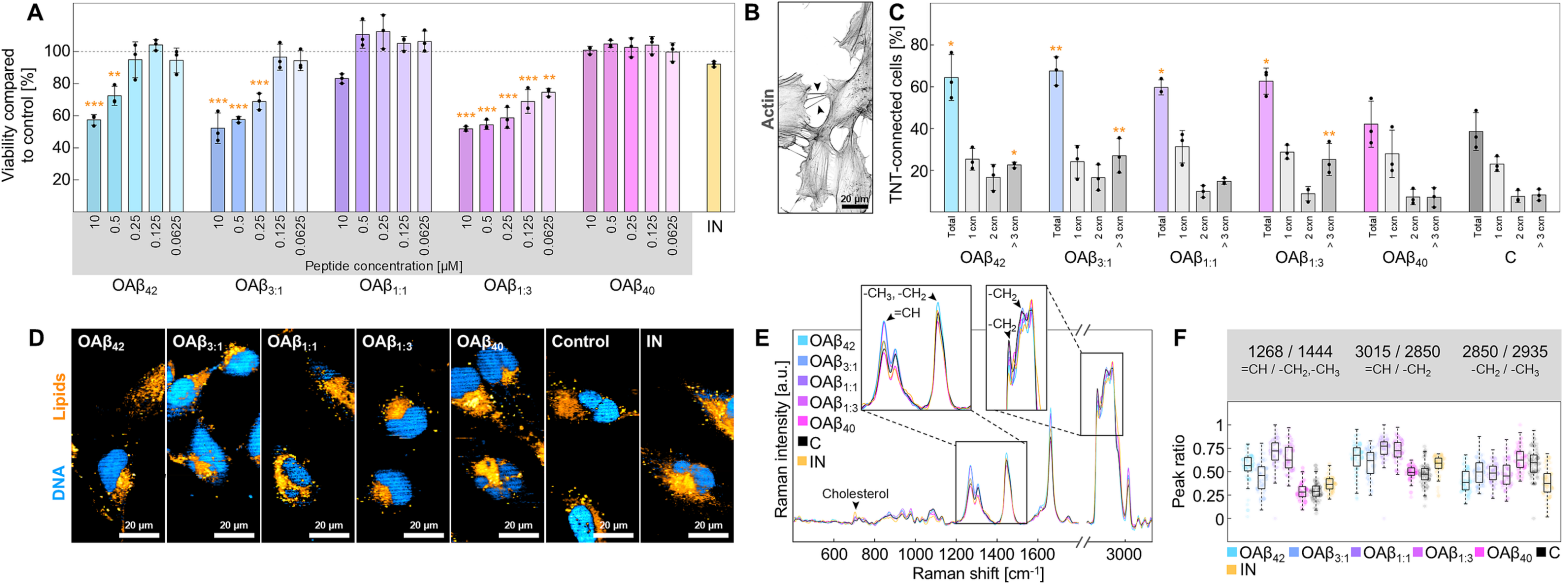
Cell viability and functionality of microglia. **(A)** Viability of microglia after treatment with varying concentrations of OAβ_42_, OAβ_3:1_, OAβ_1:1_, OAβ_1:3_, or OAβ_40_ (*n* = 3). Viability was normalized to the control (medium). Statistical significance is indicated compared to the control. **(B)** Actin staining of microglia with arrowheads indicating examples of TNTs. **(C)** Results of the TNT measurements after exposition to 10 μM OAβ_42_, OAβ_3:1_, OAβ_1:1_, OAβ_1:3_, OAβ_40_, or medium (control) (*n* = 3). Statistical significance is indicated compared to the control. **(D)** Raman images showing DNA (blue) and lipids (orange) of microglia treated with 10 μM OAβ_42_, OAβ_3:1_, OAβ_1:1_, OAβ_1:3_, OAβ_40_, medium (control), or LPS + IFN-*γ*. **(E)** Raman spectra of lipid droplets of microglia after treatment with 10 μM OAβ_42_, OAβ_3:1_, OAβ_1:1_, OAβ_1:3_, OAβ_40_, or medium (control). The zoomed-in sections and arrowheads mark typical lipid peaks. **(F)** Peak ratio analysis for peaks of interest. *p < 0.05, **p < 0.01, ***p < 0.001, C, control; IN, inflamed model; cxn, connection(s).

Similar trends regarding cell viability were also observed in microglia (Figure 5A). While the viability of microglia after exposure to 10 μM OAβ_40_ or pro-inflammatory stimuli (LPS + IFN-*γ*) was not significantly reduced (100.80 ± 2.38% and 92.12 ± 1.72%), OAβ_42_, OAβ_3:1_, and OAβ_1:3_ showed significant concentration-dependent toxicity (57.41 ± 3.29%, 52.19 ± 9.50%, and 51.83 ± 1.42%), except for OAβ_1:1_ (83.13 ± 2.93%). Concerning TNT formation, 38.54 ± 9.07% of cells of the control were connected by at least one TNT, 7.41 ± 2.87% were linked by two TNTs and 8.17 ± 2.74% were joined by more than three TNTs (Figures 5B,C); similar observations were also made for OAβ_40_ treated cells. However, OAβ_42_, OAβ_3:1_, and OAβ_1:3_ treatment significantly increased the total number of TNT-connected cells (64.44 ± 11.00%, 67.56 ± 6.82%, and 62.66 ± 6.29%), as well as the portion of cells connected by three or more TNTs (22.55 ± 1.37%, 26.97 ± 8.25%, and 25.18 ± 7.69%). Moreover, Raman imaging revealed an increased ratio of unsaturated/saturated bonds in lipid spectra in microglia treated with mixed oligomers or OAβ_42_ (Figures 5D–F), contrasting results obtained from the analysis of astroglial lipid droplets (Figures 4E,F). Furthermore, Raman spectra of microglial lipids droplets displayed a slightly lower chain length after treatment with OAβ_42_, mixed oligomers, and LPS + IFN-*γ*. An elevated cholesterol peak was only detected after LPS + IFN-γ exposure.

### 3.3 Impact of OAβ_42_ and OAβ_1:3_ and on a model of the BBB

**Figure 6:**
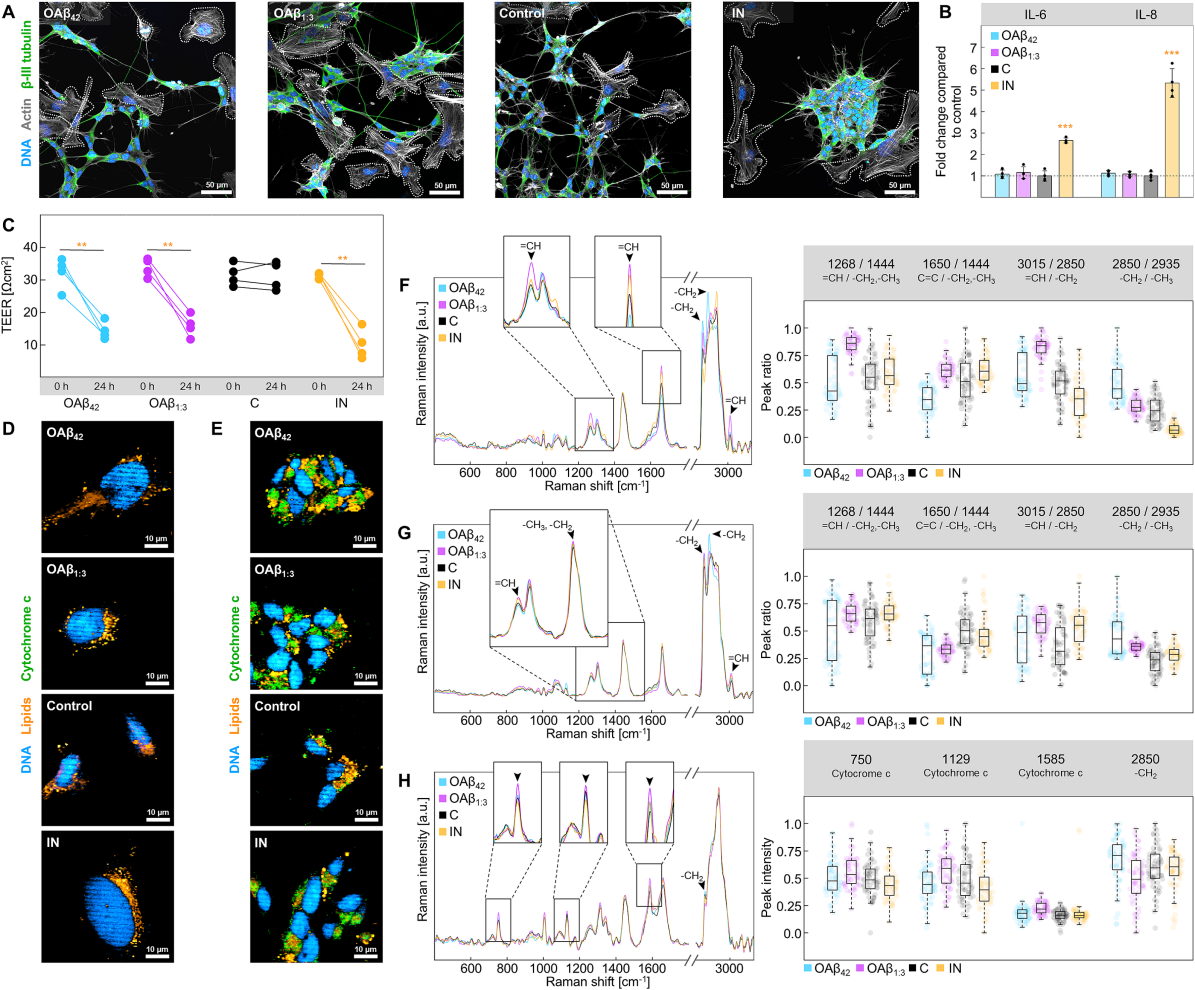
Analysis of the BBB model. **(A)** Staining of DNA (blue), actin (gray) and β-III tubulin (green) in the basal compartment of the BBB model after treatment with 10 μM OAβ_42_, 10 μM OAβ_1:3_, medium + PBS (control), or LPS + IFN-γ. Microglia are circled with dashed lines. **(B)** Results of ELISA for IL-6 and IL-8 after treatment with 10 μM OAβ_42_, 10 μM OAβ_1:3_, medium + PBS (control), or LPS + IFN-γ (*n* = 3) showing the fold change compared to the control. **(C)** Results of TEER measurement for exposure to 10 μM OAβ_42_, 10 μM OAβ_1:3_, medium + PBS (control), or LPS + IFN-γ (*n* = 4). Statistical significance is indicated compared to the 0 h timepoint. Raman images of **(D)** microglia and **(E)** neurons in the basal compartment of the BBB model showing DNA (blue), lipids (orange), and cytochrome c (green) after exposure to 10 μM OAβ_42_, 10 μM OAβ_1:3_, medium + PBS (control), or LPS + IFN-γ. Raman spectra of lipid droplets of microglia **(F)**, lipid droplets of neurons **(G)** and cytochrome c of neurons **(H)** are displayed on the left, with zoomed-in sections showing peaks of interest. Corresponding peak ratio or intensity analysis is shown on the right side. *p < 0.05, **p < 0.01, ***p < 0.001, C, control; IN, inflamed model.

Observations of toxic effects in single cells sparked a further investigation of the impact of OAβ_42_ and OAβ_1:3_ on a human-based *in vitro* model of the BBB, composed of endothelial cells in the apical compartment, astroglia lining the basal side of an insert membrane and neurons and microglia in the basolateral compartment (Figure 1). After treatment with OAβ_42_, OAβ_1:3_ or LPS + IFN-γ, fluorescence staining and ELISA (Figures 6A,B) uncovered striking differences between LPS + IFN-γ and Aβ oligomer-treated models. Precisely, microglial and neuronal morphology of OAβ_42_- and OAβ_1:3_-treated models resembled the control, showing intertwined microglia and neurons, as well as extended neurites. Conversely, neurite length was reduced in models treated with LPS + IFN-γ, with microglia circling neurons. Secretion of pro-inflammatory cytokines IL-6 and IL-8 was also strongly increased in these models, whereas no prominent change was observed for samples treated with OAβ_42_ and OAβ_1:3_. TEER was significantly reduced in models treated with OAβ_42_, OAβ_1:3_, as well as LPS + IFN-γ from 32.13 ± 4.83 Ωcm^2^ to 14.36 ± 2.73 Ωcm^2^, 33.83 ± 2.84 Ωcm^2^ to 15.84 ± 3.41 Ωcm^2^, and 31.47 ± 0.87 Ωcm^2^ to 10.11 ± 4.61 Ωcm^2^, respectively (Figure 6C), indicating barrier disintegration in each case. While Raman images of microglia resembled the data acquired from microglial monoculture (Figures 5D, 6D), we identified distinct lipid species in neurons (Figure 6E), which were not detectable in monoculture (Supplementary Figure S4). Further differences between treatments with OAβ_1:3_, OAβ_42_, and LPS + IFN-γ were revealed using peak ratio analysis. In microglia, OAβ_1:3_ application led to a shift of lipid spectra toward unsaturated lipids, which was absent for OAβ_42_. Instead, OAβ_42_ induced an increase in lipid chain length, which did not occur to the same extent for OA*β*_1:3_. LPS + IFN-γ treated models displayed only a slight shift toward unsaturated lipids with a simultaneous decrease in chain length (Figure 6F). Lipid spectra acquired from neurons displayed fewer differences, and a trend toward unsaturated or saturated lipids was not as clear; however, chain length was again longest after OAβ_42_ treatment (Figure 6G). Furthermore, investigation of neuronal cytochrome c revealed elevated levels of cytochrome c, as well as a decreased lipid peak intensity, both of which were strongest for treatment with OAβ_1:3_ (Figure 6H).

## 4 Discussion

### 4.1 Role of Aβ_40_ on Aβ_42_ fibrillation and structural characterization of mixed oligomers

As protein accumulation is a central characteristic of Aβ-induced pathology, evaluating aggregation kinetics of differing Aβ_42_:Aβ_40_ ratios is crucial to investigate their toxicity as co-aggregation of Aβ_42_ and Aβ_40_ leads to the formation of oligomers containing both isoforms (Vadukul et al., 2020). Our results clearly indicate a sequential reduction of the aggregation speed of Aβ_42_ after the addition of Aβ_40_, implying an inhibitory effect of the shorter peptide variant on Aβ_42_ fibrillation (Figure 2A) (Chang and Chen, 2014; Braun et al., 2022). Thus, considering the direct impact of aggregate stability, size, and conformation on Aβ toxicity, the observed decrease of plateau height and aggregation speed may contribute to understanding the toxicity of co-aggregated Aβ_42_ and Aβ_40_ (Cizas et al., 2010; Chang and Chen, 2014; Vadukul et al., 2020). While prior studies mostly focused on pure oligomers, the formation of mixed oligomers, and especially the ratio of Aβ_42_:Aβ_40_, seems to be a crucial factor in neurodegeneration and overall AD-related pathophysiology (Chang and Chen, 2014; Kuperstein et al., 2010; Kwak et al., 2020). The strikingly different aggregation kinetics motivated us to delve further into the structural characterization and composition of Aβ aggregates using AFM and Raman spectroscopy. Our aggregation protocols for monomers, pure and mixed oligomers, as well as fibrils, yielded sizes matching the described range in literature (Figures 2B,C) (Ungureanu et al., 2016; Cizas et al., 2010; Manassero et al., 2016). Despite being a valuable tool for analyzing Aβ aggregates due to its sensitivity to secondary structure, Raman spectroscopy (Figures 2D–G) is rarely used in the context of neurodegenerative diseases. Existing Raman spectroscopic studies have mostly been confined to the examination of fibrillar Aβ (Flynn and Lee, 2018). Notably, Yu et al. (2018) investigated the Aβ aggregation kinetics using surface-enhanced-Raman-spectroscopy (SERS), a technique designed to amplify the Raman signal. However, using SERS affects the location and intensities of Raman peaks, impeding interpretation and making it unsuitable for assessing aggregate conformation. Hence, we sought to explore the potential of Raman spectroscopy in the analysis of amyloid aggregates using drop-coating deposition Raman spectroscopy (DCDRS), entrapping biomolecules in a hydrated environment while decreasing detection limits (Peters et al., 2016; Ortiz et al., 2006). Through cross-correlation analysis, we revealed changes in the secondary structure of beta-amyloid owing to the typical conversion of unordered regions and α-helices to β-sheets (Mallesh et al., 2023). Interestingly, the analysis also uncovered previously unidentified shifts in the Raman spectra. For instance, the amide II band, typically faint due to low scattering effects, was distinguishable for monomeric Aβ_42_ and faded during Aβ aggregation, indicating an increased rigidity in the amide backbone (Mensch et al., 2017). The decrease in relative intensity of amino acid side chains, such as phenol rings or acyl chains, further suggested constriction of molecular vibrations during this process. We pinpointed these sequential changes in amyloid structure using peak ratio analysis, demonstrating reproducible trends from monomers to fibrils. Overall, Raman spectroscopy offers a straightforward approach to differentiating aggregate types since DCDRS requires minimal sample preparation as opposed to AFM. However, distinguishing aggregate types depending on Aβ_42_:Aβ_40_ ratio proved challenging, and further research is needed to address this question.

### 4.2 Effect of Aβ_42_ fraction in mixed oligomers on the endothelium

To examine the effects of Aβ oligomers depending on the Aβ_42_:Aβ_40_ ratio, we treated AD-relevant brain cells with pure and mixed oligomers. Importantly, the peptide concentrations used in this study were much higher than physiological levels of soluble Aβ found in brain tissue of AD patients (Roberts et al., 2017). However, such high concentrations are considered necessary to stimulate immortalized cell lines (McCarthy et al., 2016). As main constituents of the BBB, we initially analyzed the cytotoxic effects of pure and mixed oligomers on endothelial cells: only OAβ_42_ and OAβ_1:3_ significantly decreased viability and displayed dose-dependent toxicity (Figure 3A). These findings were confirmed by our investigation of barrier integrity, which showed significant decreases of TEER only after exposure to OAβ_42_ and OAβ_1:3_. This effect has been described for OAβ_42_, but not for OAβ_1:3_ (Figure 3B) (Parodi-Rullán et al., 2020). Further, reduction of angiogenesis has also been depicted in prior reports and was found in our study after exposure to OAβ_42_, as reported, and OAβ_1:3_ (Figures 3C,D) (Parodi-Rullán et al., 2020; Paris et al., 2004). The fact that vascular amyloid deposits mainly constitute Aβ_40_, combined with the detrimental effects observed in OAβ_1:3_ treated endothelial cells, calls for further studies investigating the role of these oligomers for BBB breakdown (Qi and Ma, 2017).

### 4.3 Mitochondrial dysfunction in neuronal cells induced by Aβ oligomers

In case of neuronal cells, our results show a decrease in viability and neurite outgrowth upon exposure to OAβ_42_, consistent with previous reports (Figures 4A–C) (Zhang et al., 2022; Petratos et al., 2008; Krishtal et al., 2017). Equally detrimental effects were observed for each mixed oligomer, aligning with studies by Kuperstein et al. (2010) and Kwak et al. (2020), the latter showing that the Aβ_42_:Aβ_40_ ratio drives tau pathology in neurons. Thus, our results underscore the importance of the Aβ_42_:Aβ_40_ ratio for AD-associated pathophysiology. Analysis of cytochrome c mRNA levels indicated enhanced expression of cytochrome c after treatment with OAβ_42_ and OAβ_1:3_, amplifying cell death (Chandra et al., 2002). Additionally, Raman imaging of neurons (Supplementary Figure S4) showed that all oligomers, except OAβ_40_, increased cytochrome c-related Raman peaks, further indicating cytochrome c accumulation. Therefore, our results implicate mitochondrial dysfunction induced by both OAβ_42_ and oligomers containing only minor amounts of Aβ_42_, such as OAβ_1:3_. These effects may stem from *N*-methyl-d-aspartate (NMDA) receptor overactivity caused by activation by Aβ oligomers (Gao et al., 2007; Li et al., 2011).

### 4.4 Cellular and metabolic changes of glial cells following Aβ oligomer exposure

Due to their essential contribution to neurodegeneration and based on the previously reported severe effect of OAβ_42_, we investigated the effects of differently mixed Aβ oligomers on astroglia (Abramov et al., 2004; Hou et al., 2011). We were able to show prominent effects of such mixed oligomers on these cells, mirroring the results of the viability assay on neurons (Figure 4D). Raman analysis indicated a shift in the composition of astroglial lipid droplets toward saturated lipids instigated by all oligomers except OAβ_40_ (Figures 4E,F). This inclination is especially interesting, considering astroglia-promoted neurodegeneration via secretion of saturated fatty acids (Guttenplan et al., 2021). Hence, our findings reinforce the hypothesis that Aβ oligomers only containing minor amounts of Aβ_42_ may be equally toxic as pure Aβ_42_ oligomers. Besides astroglia, microglia are also strongly linked to AD due to their substantial contribution to neuroinflammation (de Dios et al., 2023). Additionally, especially activated APOE (apolipoprotein E) positive microglia play an important role in Aβ plaque formation and compaction (Kaji et al., 2024; Spangenberg et al., 2019). Our investigation not only confirmed the already reported toxicity of OAβ_42_ but also uncovered the severe cytotoxic effects of OAβ_3:1_ and OAβ_1:3_ (Figure 5A) (Pan et al., 2011). The observed proliferative effect (viability above >100% compared to control) after OAβ_40_ treatment may be linked to a physiological response of the microglia to clear Aβ, albeit this effect was not significant (Fruhwürth et al., 2024). Interestingly, Aβ oligomers also induce the formation of TNTs, serving as a transportation system for Aβ in the central nervous system (Zhang et al., 2021). Previous studies have reported increased TNT-based connections between microglial cells when exposed to Aβ or other toxic peptides like *α*-synuclein (Chakraborty et al., 2023; Dilna et al., 2021). Our study showed not only an increase in microglia connected with one TNT across the treatment with all oligomers – except OAβ_40_ – but also a substantial number of cells linked by multiple TNTs (Figures 5B,C). In fact, a previous study found that Aβ_42_ oligomers are incorporated into endo-lysosomal vesicles, which subsequently transport Aβ between neurons via TNTs (Dilna et al., 2021). Furthermore, other pathological proteins such as prions are also transported inside endo-lysosomal vesicles through TNTs between cells (Zhu et al., 2015). Therefore, it is likely that the TNTs observed in this study served a similar purpose. In addition to TNT formation, microglial lipid metabolism is associated with AD, and we detected an increase in unsaturated lipids in microglial lipid droplets, contrasting the results obtained with astroglia (Figures 5D–F). Interestingly, the lipid profile of LPS + IFN-*γ*-stimulated microglia markedly differed from cells exposed to oligomeric Aβ, implying different inflammatory cellular responses. These observations are also supported by the viability assay, which showed no significant decrease upon LPS + IFN-*γ* treatment. Conversely, Aβ oligomers induced a different pathological state, which is highlighted by Raman analysis of lipid droplets. Relative lipid chain length was decreased for cells treated with mixed oligomers, OAβ_42_, as well as LPS + IFN-*γ*, however, a strong rise in unsaturated lipids was only observed for mixed oligomers and OAβ_42_. These alterations in lipid droplet composition toward unsaturated lipids have been identified as a hallmark of inflammation, suggesting production of pro-inflammatory lipids such as arachnoid acid and prostaglandins (Czamara et al., 2017; Chausse et al., 2021). Moreover, the increase of unsaturation is possibly also linked to APOE, which induces abnormal accumulation of unsaturated fatty acids in lipid droplets in AD (Sienski et al., 2021). APOE also facilitates the uptake and compaction of Aβ in the microglial endo-lysosomal system, which leads to the formation of indigestible Aβ aggregates which are released to the surrounding and contribute to plaque growth (Kaji et al., 2024). Thus, APOE not only influences abnormal lipid metabolism but also drives amyloidosis, and future studies should further address the link between the two effects. Meanwhile, LPS + IFN-γ-stimulated cells displayed only a moderate rise in unsaturation and an increase of cholesterol, which was not detected in mixed oligomers and OAβ_42_-treated cells. Although underlining the differences in the induced inflammatory state, this finding is unexpected as raised levels of cholesterol in microglia are considered a feature of neurodegenerative diseases (Zareba and Peri, 2021; Muñoz Herrera and Zivkovic, 2022). Presumably, the relative increase in cholesterol in Aβ oligomer-treated cells was below the detection limit, while exposure to a strong pro-inflammatory stimulus like LPS + IFN-*γ* caused a higher relative cholesterol increase. Importantly, the results presented here only encompass the treatment of cultured cells with soluble Aβ oligomers, but not an analysis of intracellular formation or trafficking of these oligomers. Presumably, amyloid precursor protein (APP) is consecutively cleaved by β-secretase and γ-secretase at the lysosomal membrane, leading to the accumulation of Aβ inside lysosomes and subsequent secretion into the extracellular compartment (Tan and Gleeson, 2019). Furthermore, the lysosomal acidic pH promotes aggregation of Aβ and formation of Aβ oligomers already before secretion, causing severe intracellular damage (Schützmann et al., 2021). The role of APOE and lipoprotein flux is particularly interesting in this context, as endocytosis of lipoproteins into lysosomes has been shown to serve as seeding platform for Aβ and to promote plaque growth (Kaji et al., 2024). Therefore, future research investigating Aβ_42_:Aβ_40_-ratio-dependent intracellular formation of oligomers, their trafficking and effects may complement the findings presented in this study.

### 4.5 Differential effects of mixed Aβ oligomers on BBB integrity and microglia activation

Overall, these monoculture-based studies on endothelial cells, neurons, astroglia, and microglia illustrate the individual effects of mixed Aβ oligomers in the different brain cell types. In each experiment, OAβ_1:3_ exposition was equally detrimental to cell viability and function as OAβ_42_ oligomers, and – in the case of endothelial cells – OAβ_1:3_ was the only mixed oligomer type showing similar toxicity as OAβ_42_. Due to their inherent structural differences, these two oligomer types possibly possess different effects on cells and were, therefore, selected as candidates for a comprehensive evaluation. By extending our study to an *in vitro* model of the BBB, encompassing all investigated cell types, we further explored differences between OAβ_42_ and OAβ_1:3_ toxicity. We observed a significant drop in TEER after 24 h of exposure to OAβ_42_, OAβ_1:3_, and LPS + IFN-γ, pointing to barrier dysfunction in each case (Figure 6C). Additionally, the absolute decrease of TEER induced by OAβ_42_ and OAβ_1:3_ was more severe than in endothelial cell monocultures. Our observations also align with reported damaging effects of inflammatory stimuli on brain endothelial barrier integrity (Hu et al., 2019). These detrimental effects may lead to both direct damage and the induction of inflammatory responses, which have been reported to shift the phenotype of resting microglia into an activated state (Caldeira et al., 2017). Specifically, literature suggests an amoeboid microglial morphology upon activation, while resting microglia are rather ramified (Wendimu and Hooks, 2022). However, the activation of microglial cells is a highly complex process dependent on various parameters (Gao et al., 2023). In our study, analysis of microglial morphology in the BBB model’s basal compartment revealed differences between oligomer treatment and LPS + IFN-*γ* stimulation, further suggesting distinctly different immunomodulatory mechanisms (Figure 6A). Accordingly, IL-6 and IL-8 secretion was strongly increased for LPS + IFN-γ treated models but rose only slightly after oligomer exposition (Figure 6B). While these results support both the concept of complex microglial phenotypes and the hypothesis of a dysfunctional microglial phenotype upon stimulation with Aβ, they contrast with evidence specifying an increase of pro-inflammatory interleukin secretion (Franciosi et al., 2005; Kiraly et al., 2023). To gain further insight into changes in cell metabolism, we performed Raman imaging on neurons and microglia (Figures 6D–H). Regarding microglia, the notable rise in the relative amount of unsaturated lipids within lipid droplets triggered by OAβ_1:3_ may be linked to involvement in eicosanoid metabolism (Farmer et al., 2020; Khatchadourian et al., 2012). This circumstance is especially interesting, considering free arachidonic acid levels and prostaglandin E2 levels are elevated in AD (Yin, 2023). Conversely, OAβ_42_ treatment primarily induced an increase of chain length, implying a higher abundance of long-chained lipids in lipid droplets. Notably, apart from polyunsaturated lipids such as prostaglandins, such long-chained saturated lipids are also involved in pro-inflammatory processes mediated by glial cells (Gupta et al., 2012). Moreover, LPS + IFN-γ did not strongly alter the ratio of unsaturated to saturated lipids but decreased relative chain length, further suggesting a different pro-inflammatory mechanism as opposed to oligomeric Aβ. Our findings also reveal the presence of lipids in neurons, which was not observed in monoculture, thereby supporting evidence of lipid transfer between cells in co-culture (Ralhan et al., 2021). Qualitative differences were not as pronounced as in microglia, yet again, OAβ_42_ led to accumulation of lipids with higher relative chain length, whereas OAβ_1:3_ induced enrichment of unsaturated lipids. Interestingly, both phenomena may be evoked by a stress reaction of neurons. Since polyunsaturated lipids are especially vulnerable to peroxidation caused by reactive oxygen species, neurons sequester them into lipid droplets (Bailey et al., 2015). Contrarily, saturated long-chain fatty acids are reportedly highly toxic, alleviated by deposition in lipid droplets, underscoring the complex function of lipid droplets in neurons (Ackerman et al., 2018). Our findings using Raman microscopy also include slight increases in reduced cytochrome c intensities and a reduction of the lipid peak intensity in neurons, particularly associated with OAβ_1:3_. These results indicate defects in the respiratory chain and lipid peroxidation, respectively, the latter of which suit the notion of unsaturated lipid rescue into lipid droplets (Abramczyk et al., 2022; Russo et al., 2019).

A possible explanation for the differential biochemical composition of BBB-associated microglia after OAβ_42_ and OAβ_1:3_ treatment as well as the pronounced toxicity of OAβ_42_ and OAβ_1:3_ on endothelial cells and microglia monocultures may be found in the results of the ThT aggregation assay: it demonstrated that Aβ_42_ is more prone to *in situ* fibrillation, while Aβ_1:3_ forms smaller stable aggregates. Thus, this points toward a dual toxicity of the different oligomeric ratios of Aβ_42_:Aβ_40_ – *in situ* fibrillation of mainly OA*β*_42_, and stabilization of low-Aβ_42_-high-Aβ_40_ oligomers, like OAβ_1:3_, by their Aβ_40_ content (Chang and Chen, 2014; Krishtal et al., 2017). Taken together, stable OAβ_1:3_ is not only similarly toxic as OAβ_42_, but these oligomers also seem to cause toxicity via a different mechanism than OAβ_42_. Furthermore, we observed a less pronounced toxicity of OAβ_42_ and OAβ_1:3_ in neurons and astroglia, which in turn suggests that endothelial cells and microglia are especially prone to Aβ_42_:Aβ_40_-ratio-dependent toxicity. Therefore, this study proves that the Aβ_42_:Aβ_40_ ratio in oligomers strongly influences cell metabolism and functionality, which is especially crucial considering that Aβ oligomers only containing minor amounts of Aβ_42_ are formed at the very beginning of AD. Hence, the overall evidence presented in our study clearly questions the Aβ_42_ oligomer-focused research in the field of AD and reveals a toxicity of Aβ oligomers, which has been previously overlooked.

## Data Availability

The raw data supporting the conclusions of this article will be made available by the authors, without undue reservation.

## Glossary

Aβ: beta-amyloid
AD: Alzheimer’s disease
AM II: amide II
APOE: apolipoprotein E
APP: Amyloid Precursor Protein
BBB: blood–brain barrier
BDNF: brain-derived neurotrophic factor
C: control
COX: cytochrome c oxidase
CXN: connection(s)
DCDRS: drop-coating deposition Raman spectroscopy
DNA: desoxyribonucleic acid
ELISA: Enzyme-linked-immunosorbent-assay
FAβ_42_: fibrillary amyloid-beta 1–42
FCS: fetal calf serum
IFN-γ: Interferon-gamma
IN: inflamed model
LPS: Lipopolysaccharide
MAβ_42_: monomeric amyloid-beta 1–42
mRNA: messenger ribonucleic acid
NMDA: *N*-methyl-d-aspartate
OAβ_40_: oligomeric amyloid-beta 1–40
OAβ_1:3_: oligomeric amyloid-beta 1–42 and 1–40 mixed 1:3
OAβ_1:1_: oligomeric amyloid-beta 1–42 and 1–40 mixed 1:1
OAβ_3:1_: oligomeric amyloid-beta 1–42 and 1–40 mixed 3:1
OAβ_42_: oligomeric amyloid-beta 1–42
PBS: phosphate buffered saline
PBS-T: phosphate buffered saline +0.05% Tween 20
PHE: phenylalanine
RNA: ribonucleic acid
RT-qPCR: Real-time quantitative polymerase chain reaction
SERS: surface-enhanced-Raman-spectroscopy
TEER: Transepithelial electrical resistance
THT: Thioflavin T
TNT: Tunneling nanotube
TYR: tyrosine
