# Supplementary material for: The Aβ42:Aβ40 ratio modulates aggregation in beta-amyloid oligomers and drives metabolic changes and cellular dysfunction
Source: Front Cell Neurosci. 2024 Dec 5;18:1516093. doi: 10.3389/fncel.2024.1516093 (PMC11664223; doi:10.3389/fncel.2024.1516093)
Supplement: Supplementary file 1 [file Data_Sheet_1.pdf]

Supplementary Material

1 Supplementary Figures and Tables

1.1 Supplementary Figures

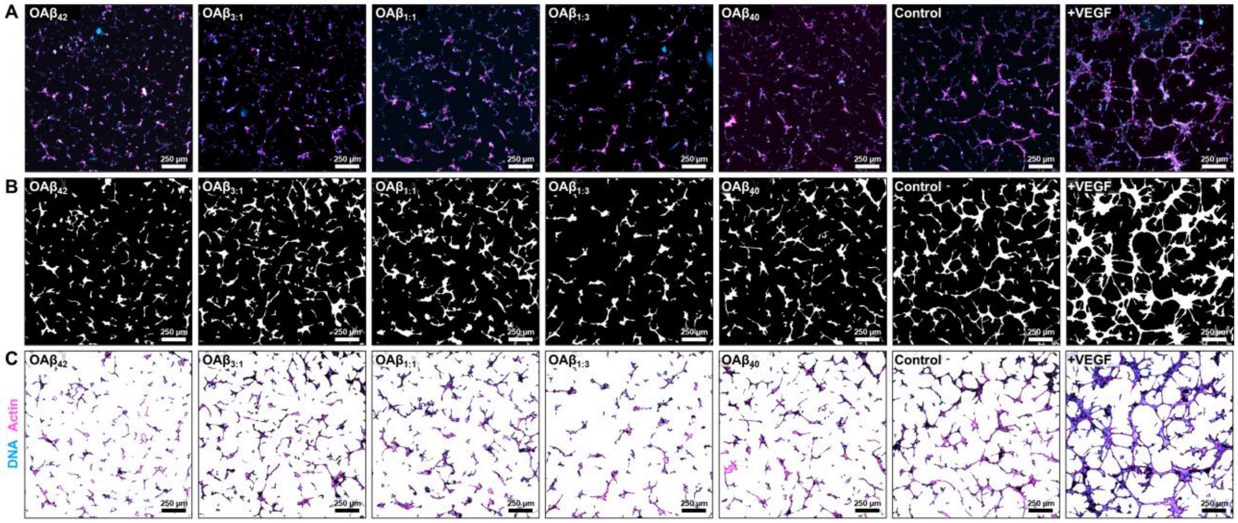

Supplementary Figure 1: Evaluation of angiogenesis. (A) Staining of DNA (blue) and actin (pink) for quantification of tube formation by endothelial cells after 20 hours exposure to OAβ_{42}, OA$\beta_{3:1}$, OA$\beta_{1:1}$, OA$\beta_{1:3}$, OAβ_{40}, control, or VEGF. **(B)** Binary images of vessel networks generated using the actin fluorescence images which were processed by the REAVER script in Matlab **(C)** Overlay of fluorescence images with binary images.

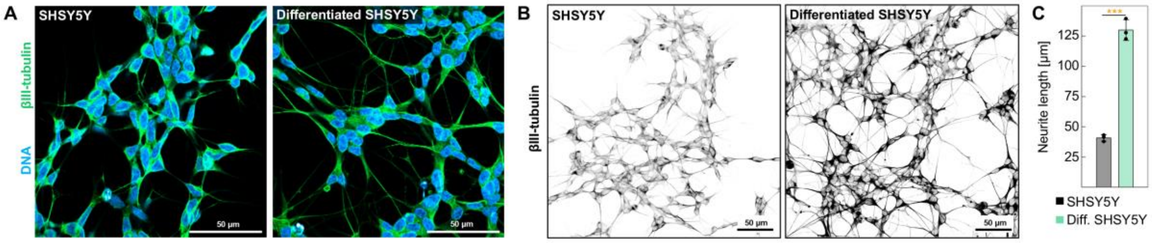

Supplementary Figure 2: Differentiation of SHSY5Y cells. (A) DNA (blue) and βIII-tubulin (green) staining for SHSY5Y cells and differentiated SHSY5Y cells at 40x magnification. **(B)** βIII tubulin staining for SHSY5Y cells and differentiated SHSY5Y cells at 20x magnification, showing network formation between cells. **(C)** Neurite length measurement shows significantly increased neurites for differentiated SHSY5Y cells (n = 3). Statistical significance is indicated by * ($p<0.05$), ** ($p<0.01$), and *** ($p<0.001$).

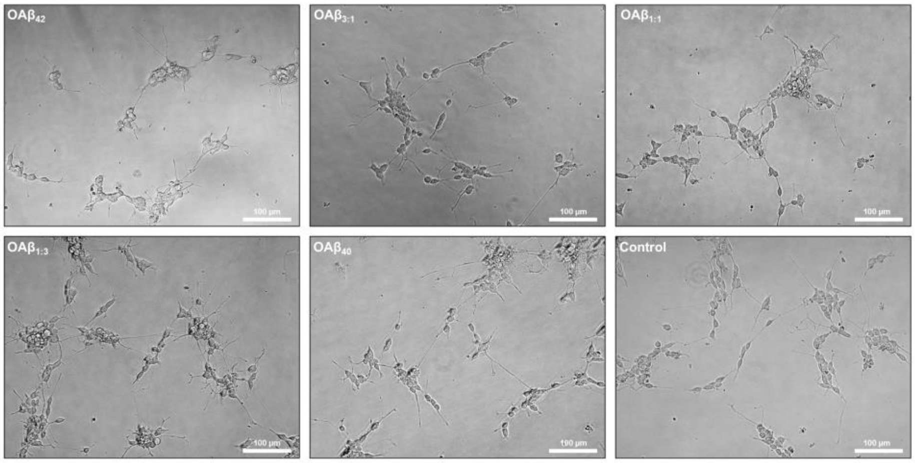

Supplementary Figure 3: Neurite outgrowth assay. Exemplary phase contrast images of neurons in the neurite outgrowth assay after incubation with $OA\beta_{42}$, $OA\beta_{3:1}$, $OA\beta_{1:1}$, $OA\beta_{1:3}$, $OA\beta_{40}$ or medium.

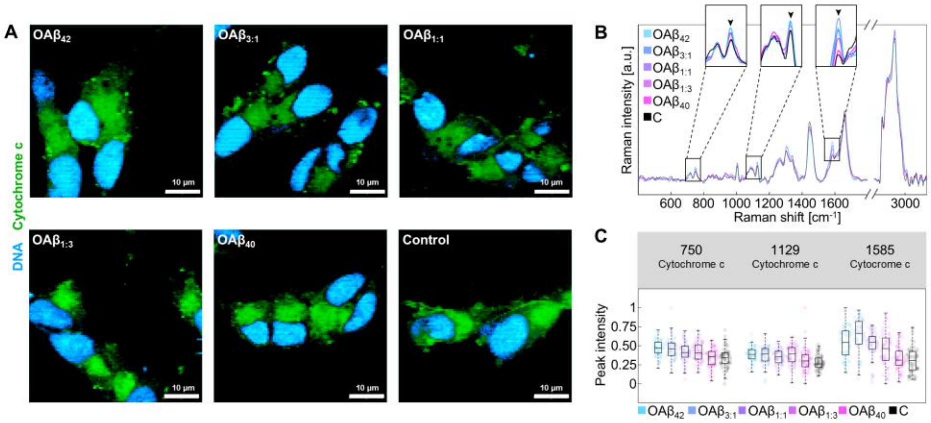

Supplementary Figure 4: Raman analysis of differentiated SHSY5Y cells. (A) Raman images showing DNA (blue) and cytochrome c (green) of cells treated with $OA\beta_{42}$, $OA\beta_{3:1}$, $OA\beta_{1:1}$, $OA\beta_{1:3}$, $OA\beta_{40}$, or medium (control). **(B)** Raman spectra of cytochrome c of treated and control neurons. The zoomed sections and arrowheads mark typical reduced cytochrome c peaks. **(C)** Peak intensities for peaks of interest. C = control.

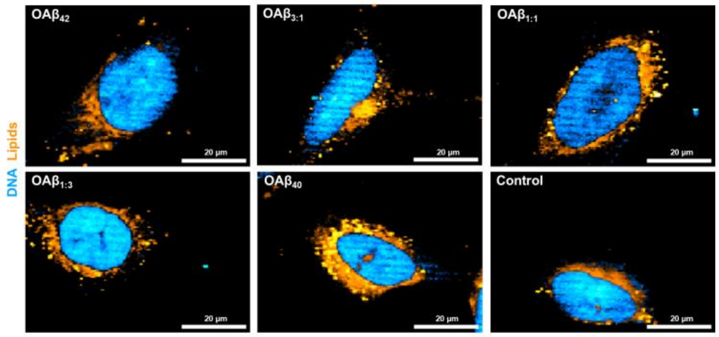

Supplementary Figure 5: Raman analysis of astroglia. Raman images showing DNA (blue) and lipids (orange) of astroglia after incubation with $OA\beta_{42}$, $OA\beta_{3:1}$, $OA\beta_{1:1}$, $OA\beta_{1:3}$, $OA\beta_{40}$ or medium (control).

1.2 Supplementary Tables

**Peaks [cm^{-1}]**	**Assignment**	**References**
702	Cholesterol	Movasaghi et al., 2007
750	Cytochrome c	Brahze et al., 2012
1129	Cytochrome c	Brahze et al., 2012
1240	β-sheet	Kuhar et al., 2021; Rygula et al., 2013
1268	=CH deformation	Czamara et al., 2015; Jamieson et al., 2018
1307	α-helix	Kuhar et al., 2021; Mensch et al., 2017
1447	$-CH_2$, $-CH_3$ deformation	Rygula et al., 2013
1555	Amide II	Mensch et al., 2017
1584	Cytochrome c	Brahze et al., 2012
1607-1615	Tyrosine, phenylalanine	Fonseca et al., 2019
1650	C=C stretching	Czamara et al., 2015; Jamieson et al., 2018
1671	β-sheet	Kuhar et al., 2021; Movasaghi et al., 2007; Rygula et al., 2013
2850	Symmetric $-CH_2$ stretching	Jamieson et al., 2018; Movasaghi et al., 2007
2885	Asymmetric $-CH_2$ stretching	Jamieson et al., 2018; Movasaghi et al., 2007
2930-2935	Symmetric $-CH_3$ stretching	Jamieson et al., 2018; Movasaghi et al., 2007
3010	=CH stretching	Czamara et al., 2015; Jamieson et al., 2018

Supplementary Table 1: Detailed list of Raman peak assignments.

1.3 Supplementary References

Brazhe, N. A., Treiman, M., Brazhe, A. R., Find, N. L., Maksimov, G. V., and Sosnovtseva, O. V. (2012). Mapping of redox state of mitochondrial cytochromes in live cardiomyocytes using Raman microspectroscopy. *PLoS ONE* 7 (9), e41990. doi: 10.1371/journal.pone.0041990.

Czamara, K., Majzner, K., Pacia, M. Z., Kochan, K., Kaczor, A., and Baranska, M. (2015). Raman spectroscopy of lipids: a review. *Journal of Raman Spectroscopy* 46 (1), 4–20. doi: 10.1002/jrs.4607.

Fonseca, E. A., Lafetá, L., Cunha, R., Miranda, H., Campos, J., and Medeiros, H. G., et al. (2019). A fingerprint of amyloid plaques in a bitransgenic animal model of Alzheimer's disease obtained by statistical unmixing analysis of hyperspectral Raman data. *Analyst* 144 (23), 7049–7056. doi: 10.1039/C9AN01631G.

Jamieson, L. E., Li, A., Faulds, K., and Graham, D. (2018). Ratiometric analysis using Raman spectroscopy as a powerful predictor of structural properties of fatty acids. *Royal Society open science* 5 (12), 181483. doi: 10.1098/rsos.181483.

Kuhar, N., Sil, S., and Umapathy, S. (2021). Potential of Raman spectroscopic techniques to study proteins. *Spectrochimica acta. Part A, Molecular and biomolecular spectroscopy* 258, 119712. doi: 10.1016/j.saa.2021.119712.

Mensch, C., Konijnenberg, A., van Elzen, R., Lambeir, A.-M., Sobott, F., and Johannessen, C. (2017). Raman optical activity of human α -synuclein in intrinsically disordered, micelle-bound α -helical, molten globule and oligomeric β -sheet state. *Journal of Raman Spectroscopy* 48 (7), 910–918. doi: 10.1002/jrs.5149.

Movasaghi, Z., Rehman, S., and Rehman, I. U. (2007). Raman Spectroscopy of Biological Tissues. *Applied Spectroscopy Reviews* 42 (5), 493–541. doi: 10.1080/05704920701551530.

Rygula, A., Majzner, K., Marzec, K. M., Kaczor, A., Pilarczyk, M., and Baranska, M. (2013). Raman spectroscopy of proteins: a review. *Journal of Raman Spectroscopy* 44 (8), 1061–1076. doi: 10.1002/jrs.4335.
